# Sour Fruit Beers—Ethanol and Lactic Acid Fermentation in Beer Production

**DOI:** 10.3390/molecules30163358

**Published:** 2025-08-12

**Authors:** Adam Głowacki, Justyna Paszkot, Witold Pietrzak, Joanna Kawa-Rygielska

**Affiliations:** Department of Fermentation and Cereals Technology, Wrocław University of Environmental and Life Sciences, Chełmońskiego 37, 51-630 Wrocław, Poland; adam.glowacki@upwr.edu.pl (A.G.); justyna.paszkot@upwr.edu.pl (J.P.); witold.pietrzak@upwr.edu.pl (W.P.)

**Keywords:** beer, alcoholic fermentation, lactic acid fermentation, sensory analysis, fruit beer, sour beer

## Abstract

Fruit and sour beers are popular due to their unique sensory characteristics. Owing to changes in physicochemical parameters, mixed culture fermentation is a promising research area. The aim of the study was to evaluate how ethanol and lactic acid fermentation, combined with the addition of berry fruits during the beer production process, influence the physicochemical and sensory characteristics of sour fruit beers. Three worts differing in hopping system were produced: one classic sweet wort and two lacto-fermented. Strawberries or raspberries were added to the young beer. This research showed that acidification of wort, fruit addition, and limiting of hopping time had a positive effect on both technological and sensory characteristics. Despite pH differences, alcohol content in beers was similar (2.52–3.21% *v*/*v*). Production method influenced mainly lactic acid (0–2.30 g/L), pH (3.53–4.79), and glycerol (0.83–1.62 g/L) contents. Non-acidified beers had the highest dextrin (17.64–23.13 g/L) and glycerol (1.36–1.62 g/L) levels. The addition of strawberries increased phenolics (205.21–237.03 mg GAE/L), FRAP (0.82–1.17 mmol TE/L), and refreshment sensation, while raspberries mainly enhanced sensory atributes (colour, foam, fruitiness, aroma). Lactic fermentation did not show a clear effect on polyphenol content or antioxidant activity. The research offers practical insights into functional beer development, with its novelty of using mixed fermentation and fruit addition to shape characteristics.

## 1. Introduction

Beer is one of the most consumed beverages in the world. It is produced by the ethanol fermentation of beer wort made of water, malt and hops. To enrich the sensory features of beer and give it additional properties, herbs, fruit or fruit preserves are increasingly being used in beer production [1]. Raw materials influence the shaping of sensory features, increase the content of bioactive compounds, and the stability of the product [2]. Beer is a source of carbohydrates, amino acids, minerals, vitamins, and phenolic compounds. Phenols are one of the groups of compounds responsible for the antioxidative activity in beer. Most of the phenolic compounds in beer come from malt (70–80%) and hops (20–30%) [3]. However, its content in the final product is influenced by each stage of the technological process.

The beer market has evolved in recent years. This phenomenon is called the beer revolution. This is manifested in the creation of a large number of local breweries that offer a wide variety of beer styles. One of the many interesting groups among them are sour beers [4]. They are characterized by a low alcohol content, a rich bouquet of sensory features, and a refreshing taste, making them a frequent choice of consumers. The desirable sensory qualities of these beers are related to the reduction in pH achieved through lactic acid fermentation by various lactic acid bacteria (LAB) strains, the addition of lactic acid, or other products that lower pH [5].

There are several technological treatments that allow the brewing wort to be acidified. One of the methods is acidification in a kettle souring process. This method is based on carrying out the lactic acid fermentation of the wort in a brew kettle, then boiling, cooling, and transferring to a fermentation tank for inoculation with yeast cells for fermentation [6]. The wort is acidified most often until the pH is around 3.5. Such a low pH affects, among others, the solubility of proteins, which in turn has an impact on the colloidal stability of the product [7]. The high content of organic acids and low pH also increase the microbiological stability of the product [8]. The stability of beer is also influenced by polyphenol compounds. Their content reduces the degradation of iso-α-acids during product storage and protects the complex molecules present in the beer against oxidationwhich reduces the quality of the beer [9]. In addition to influencing the technological features of fermented beverages, polyphenols have several health-promoting properties. As dietary components, they protect against the development of digestive system diseases and type 2 diabetes. Moreover, they contribute to the modulation of energy metabolism [10]. Moreover, the antioxidant and anti-inflammatory effects of polyphenolic compounds belonging to the flavonoids group have been proven [11].

Most commonly additives used in beer production are berries such as raspberries (*Rubus ideaus*) and strawberries (*Fragaria x ananassa*). They are characterised by a high content of polyphenols, especially anthocyanins, which shape their antioxidant properties. Furthermore, the addition of these fruits is an interesting way to modulate the sensory characteristics of beers.

The aim of the research was to evaluate how ethanol and lactic acid fermentation, combined with the addition of berry fruits during the beer production process, influences the physicochemical and sensory characteristics of sour fruit beers. The goal was achieved by analyzing carbohydrate profiles, ethanol, glycerol, and lactic acid content in beers. Additionally, an analysis of total polyphenols content and antioxidant properties of wort and beers was performed in subsequent technological stages.

## 2. Results and Discussion

### 2.1. Basic Physicochemical Parameters

Table 1 presents the fundamental physicochemical parameters of the beers, including alcohol content (%*v*/*v*), apparent extract content (%*w*/*w*), real degree of fermentation (RDF; %), density, caloric value (kcal/100 mL), and pH. These characteristics play an important role in determining product quality and contribute to the sensory appeal [12,13].

The average alcohol content of the analyzed beers was 2.87% *v*/*v*. The highest content was recorded in the samples with the addition of strawberries (3.1% *v*/*v*), which may be due to the higher content of fermentable sugars in these fruits; this is also confirmed by the highest degree of attenuation in these sample variants. Samples with a short boiling process had a higher alcohol concentration compared to samples boiled for 60 min, both acidified and non-acidified. The reason for this may be the Maillard reaction and caramelization of sugars during long heat treatment, resulting in a reduction in the sugars available for yeast [14]. Maillard reactions are initiated by a reaction between reducing sugars and amino acids. As a result, a number of chemically diverse products are formed, which have a strong influence on the sensory properties, shelf life, and nutritional value of food. Caramelization, on the other hand, is the process of converting sugars under the influence of high temperature, which proceeds with the release of desirable aromas. As a result of these changes, the available sugar pool for yeast may be reduced [15]. One of the main parameters that characterize sour beers is the pH, which is usually 3.0–3.9 [12]. Control sample B1C, prepared without the use of LAB, had a pH equal to 4.79. The use of LAB in the production process reduced the pH to an average value of 3.76. Ciosek et al. [13] in their study used a strain of another LAB species (*Lactobacillus brevis* WLP672), the pH obtained during 48 h of lactic acid fermentation was approximately 3.9 [13]. The addition of fruit to non-acidified samples (B1S and B1R) resulted in a decrease in pH to an average value of 4.24. All beers with fruit addition had a lower pH than the control beers. This is due to the high content of organic acids, mainly citric acid, and malic acid in berries [16].

Beers with raspberries had a lower pH value than variants with strawberries. This is supported by the study by Souza et al. [17], who analyzed the chemical composition and properties of different berries. The study showed that the pH of strawberries was 3.73, while the pH of raspberries was equal to 2.86, with titratable aciditybeing equal to 1.88 and 0.86 g citric acid/100 g fruit in raspberries and strawberries, respectively [17]. Shortening of boiling time did not have an impact on the pH values of fruit beers obtained.

The apparent extract content of the samples with fruits was lower than in control samples (without fruit), likely due to both the higher alcohol content and dilution caused by the water-rich fruit. In the short-boiled samples (B3), the extract content was slightly higher than in the long-boiled samples (B2). The dilution might also contributed to the reduced caloric content observed in fruit beers. Fruit beers also exhibited reduced caloric values: raspberry beers showed the lowest (23.32 kcal/100 mL), followed by strawberry (25.82 kcal/100 mL), and beers without fruit (27.41 kcal/100 mL).

One of the key parameters in the brewing technology is the degree of attenuation, which shows the extent of fermentables utilization throughout fermentation and lagering. Beers with added fruits showed higher attenuation than those without. The highest value was observed in beers with strawberries (63.47%), followed by raspberry beers (59.51%) and control samples (57.88%). Kawa-Rygielska et al. [2] also reported increased attenuation in fruit beers (cornelian cherry), which they attributed to the presence of fermentable simple sugars in the fruit [2]. Lactic acid fermentation did not have a significant effect on the attenuation of our experimental samples. However, among sour beers, those brewed with a short boiling time showed a lower attenuation.

### 2.2. Content of Fermentation Products and Carbohydrate Profile of Beers

The results of the HPLC analysis showing the content of carbohydrates, glycerol and lactic acid in the tested beers are presented in Table 2. The compounds analyzed belong to key factors that influence the sensory attributes of the product, such as taste or mouthfeel.

In samples prepared without the application of LAB, no lactic acid was observed, indicating the lack of ability of the yeast used to produce this compound and the absence of microbial contamination of the samples [18]. In the remaining samples, lactic acid was present, with a higher content in the short-boiled samples, which may be related to the degradation of lactic acid during prolonged thermal treatment. The lowest lactic acid content was found in beers with raspberries (1.88 g/L on average), while the highest content was found in beers with strawberries (2.26 g/L on average). Shortened boiling process resulted in obtaining slightly higher content of lactic acid in beers which implies its degradation or precipitation during long heat treatment.

Acidification of the wort resulted in a lower glycerol content in the finished products. This may be related to the activity of aldehyde dehydrogenase, which increases in alkaline environments. Lower levels of glycerol in acidified beers may be the result of limited availability of the precursor (phosphodihydroxyacetone) for the glycerol synthesis pathway or its increased consumption in other metabolic processes [19]. The highest concentration of glycerol was observed in sample B1C (1.62 g/L). On average, non-acidified samples contained 1.45 g/L of glycerol, which was 0.40 g/L more than acidified ones. Glycerol plays an important role in shaping the sensory characteristics of fermented beverages. It contributes to viscosity, body fullness, density, smoothness, and roundness, key factors that influence mouthfeel. As a result, a higher glycerol content is often associated with a fuller and more balanced sensory profile [20].

Non-acidified beers were characterized by complete attenuation of disaccharides (maltose) in contrast to the other samples. On the contrary, acidified beers had a lower maltotriose content, as well as dextrins, most likely due to *Lactiplantibacillus plantarum* bacteria that exhibit extracellular amylolytic activity [21].

### 2.3. Total Phenolic Content and Antioxidant Activity

The antioxidant activity of beer is mainly due to polyphenolic compounds and melanoidins. The key phenolic compounds present in beer include epicatechin, gallic acid, protocatechuic acid, (+)-catechin, vanillic acid, ferulic acid, p-coumaric acid and syringic acid [22]. The addition of fruits can be an effective method to enhance the antioxidant activity, as well as to make its sensory characteristics more attractive [23]. Table 3 and Table 4. present the total phenolic compound (TPC) levels and antioxidant potential levels of the beers produced in this study.

Sweet wort contained 39.54 mg GAE/L of phenolic compounds. The lactic acid fermentation process increased its content by 47.36% (58.27 mg GAE/L), but this is not reflected in an increase in antioxidant potential. It is known that low pH promotes the extraction and stability of phenolic compounds, resulting in higher TPC content in acidified sweet worts, however Folin-Ciocalteu test can overestimate the phenolics content due to interference from nonphenolic compounds, such as amino acids, reducing sugars or vitamin C [24].

In our study, hopping resulted in enhancement of TPC up to 3 times. Both sour worts had a higher content of phenolic compounds (long boiled had 112.91 mg GAE/L, while short boiled had 188.55 mg GAE/L). Ethanol and lactic acid fermentation are interesting bioprocessing methods to enhance the functional properties of plant-based products by affecting the bioavailability of phenolic compounds [25]. Lactic acid fermentation influences both the quantity and the quality of bioactive compounds in food. Polyphenols are the main antioxidants in food and play a crucial role in shaping taste, smell, and color, so their content is widely studied [26].

Microbial enzymes during fermentation affect the content and composition of antioxidants. Phenolic compounds in food are found mainly in the form of glycosides or esters, rarely in free form. As a result of enzyme activity from microorganisms, glycosides are converted to aglycone forms that exhibit higher antioxidant potential [27]. *L. plantarum* is characterized by the activity of enzymes, including tannase, ferulic acid esterase, phenolic acid decarboxylase, and beta-glucosidase, leading to the formation of antioxidants during fermentation, that is, hydroxytyrosol and pyrogallol, as well as 4-vinylguaiacol and 4-vinylphenol [26].

Heat treatment is the cause of the degradation of some phenolics [22]. This is confirmed by the reduced phenolic content in the worts boiled for 60 min samples. Reducing the hopping time to 5 min resulted in a higher content of phenolic compounds. Meanwhile, melanoidins, macromolecular compounds with antioxidant properties, are formed during heating, which explains a higher antioxidant potential in these samples. Moreover, boiling promotes the formation of polyphenol-protein precipitates that are formed, which may promote some decrease in TPC [22]. Reduced heating (short-time boiled sample) did not result in significant changes in the antioxidant potential of hopped wort compared to sweet wort. For the remaining variants, an increase in antioxidant activity was observed after hopping.

In the production of sour beers, pH is an important factor that characterises the ethanol fermentation environment. Changes in pH values affect microbial growth, enzyme activity, stability, and transformation of phytocompounds, and changes in the position of hydroxyl groups. All the factors mentioned above affect antioxidant activity [27]. The first stage of ethanol fermentation in the brewing, called main fermentation, resulted in an increase in TPC by 31.44 to 56.25% (Table 4). At this step, the sample with the highest content of phenolic compound content was the sample produced by T2 technology (acidified, 60 min boiled wort), which contained 176.42 mg GAE/L phenolics. The ability to reduce iron ions after fermentation increased significantly only for beers made with acidified wort (30.43–75.00%). Ethanol fermentation, as in the case of lactic acid fermentation, caused transformations involving enzymes of microbial origin. It can affect the content of phenolic compounds by releasing them from glycosides or the plant cell wall [27]. On the other hand, a short boiling time (5 min) caused initially high content of phenols, which was subsequently reduced after fermentation. Polyphenolic compounds form complexes with proteins. During fermentation, protein precipitation occurs. The removal of these complexes during filtration may be responsible for the observed decrease in phenolic compounds [22].

In this study, fruit were added to the beers prior to the post fermentation. It was determined that post-fermentation resulted in an increase in phenolic content for non-acidified samples (B1C, B1S and B1R). Unlike in beers made based on acidified wort, an increase in phenolic content was observed after fermentation for B2S and all samples made with acidified wort made with short-boiled wort (B3C, B3S, B3R). The antioxidant activity value of the acidified control samples (B2C, B3C) did not change statistically significantly after the main fermentation, while for B1C (non-acidified) beer it decreased by 20%.

The kind of fruit used was also a factor that influenced the phenolic content. The addition of strawberries increased the phenolic content from 9.88% (B1S) to 55.99% (B2S). In beers with raspberries added, the phenolic content was lower than for the control sample by 27.54% for B1R, while for B2R and B3R no statistically significant changes were shown after the addition of raspberries compared to the respective control samples without fruits (B1C, B2C, B3C). The addition of strawberries or raspberries resulted in an increase in antioxidant potential ranging from 28.57 to 67.14%for all samples except B3R, where no statistically significant changes of this parameter were recorded compared to beer after fermentation. Fruits in beer recipes allow the beverage with bioactive compounds and increase its antioxidant activity [2,23].

The maturation process led to a decrease in the phenolic content for most samples. A statistically significant increase was recorded for B1S and B1R–both made from non-acidified wort, and for B2R. However, elevated phenolic levels in B1S and B1R did not correspond to higher antioxidant potential. Among the non-acidified variants, sample B1S (with strawberries) exhibited the highest TPC and the ability to reduce iron ions (FRAP). It also showed the highest phenolic content and antioxidant activity among all finished beers. All control samples (B1C, B2C, B3C) were classified as statistically homogeneous in terms of phenolic content. The addition of strawberries and raspberries resulted in an increase in the content of these compounds in the beer by 24.18–54.36% and 13.64–34.95%, respectively. The addition also ehnanced the antioxidant activity of finished beers compared to beers without additives (B1C, B2C, B3C). The increase in FRAP in strawberry beers ranged from 51.85 to 82.14% and in raspberry beers from 21.43 to 64.81%. The antioxidant properties of the beers produced in our experiment were lower or similar to those of beers enriched with cornelian cherry-enriched beers, as reported by Kawa-Rygielska et al. [2].

Beers previously obtained by Kawa-Rygielska et al. [2] were characterised by a significantly elevated content of phenolic compounds (398.1 to 688.7 mg GAE/L), which may be attributed to theirgreate initial wort extract (12.44–13.21% *w*/*w*). The higher content of TPC (399–767 mg GAE/L) and FRAP (3.08–9.76 mmol/L) antioxidant activity were also characteristic of the fruit beers studied by Nardini and Garaguso [22]. However, it should be noted that all of these beers contained significantly more ethanol than those obtained in our experiment.

### 2.4. Sensory Analysis

**Figure 1:**
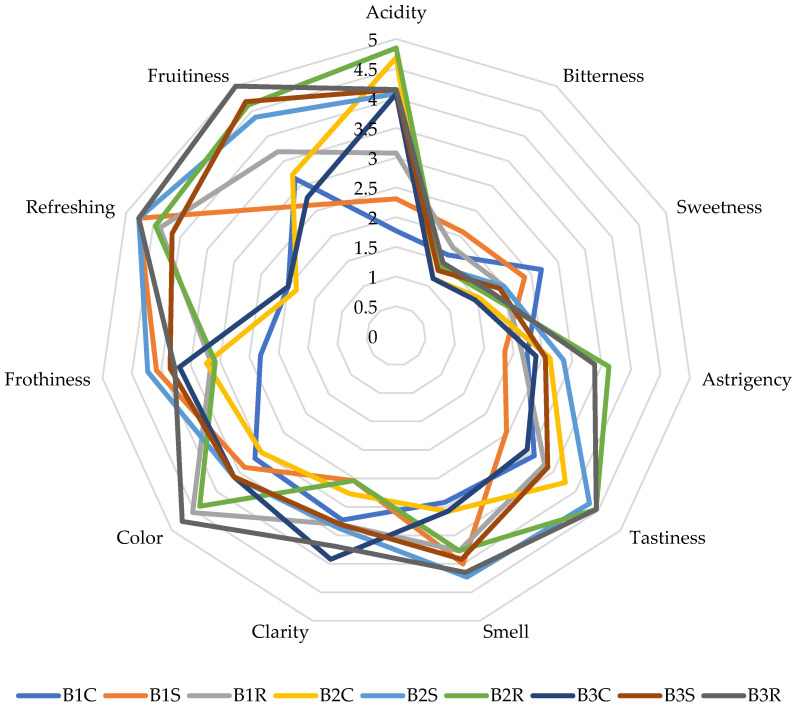
Result of the sensory analysis of beers (the full results of the sensory analysis, along with the results of the statistical analysis, are included in Supplementary Table S2).

**Figure 2:**
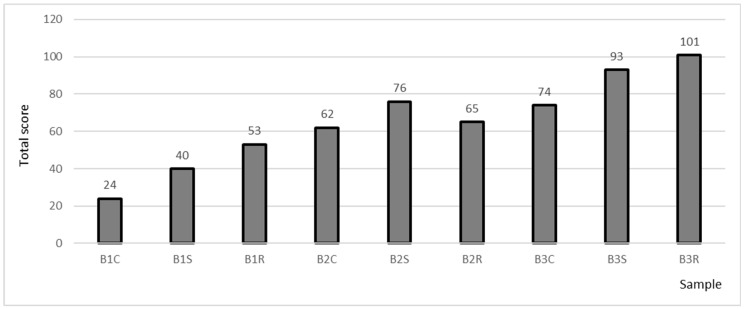
Results of the hedonic evaluation of beers.

Figure 1 and Figure 2 and Supplementary Table S2 show the results of the sensory analysis of the beers studied. Acidity, sweetness, astringency, aroma, clarity, color, frothiness, refreshment, and fruitiness were evaluated.

The testers involved in the sensory analysis of the products rated the most relevant descriptors of sour beers on a five-point scale, based on the descriptions obtained. The most intensely sour sample was B2R beer, it was also recognized as the most astringent. This was confirmed by instrumental analysis, which showed that the B2R sample had one of the lowest pH values. The B1C beer, created without the use of LAB and the addition of fruit, was described by the testers as mildly sour and mildly astringent. The addition of fruit to the unacidified beer reduced the sensation of astringency while increasing the acidity. The testers rated all acidified samples and beers with fruit as less sweet than the B1C control sample, which had the highest dextrin and maltotriose content.

The addition of fruit resulted in a marked improvement in the aroma of the beers. The samples rated the highest in terms of this parameter were B2S, B3R, and B1S. The beer with raspberries was characterized by the best palatability, and the addition of strawberries also improved the palatability of the samples. Samples with the addition of fruits were described as more refreshing, which also increases the sensory attractiveness of these products. The use of LAB in the production of the beers intensified the sensation of fruitiness. Astringency and acidity were noticeably lower in the samples with the omitted long boiling process compared to the samples with standard production technology. The omission of boiling resulted in increased fruitiness and improved clarity and color of the beers.

Figure 2 presents the results of sensory analysis of beers on a hedonic scale. The analysis showed that both acidification of the samples and the addition of fruit significantly improve the sensory qualities of beers.

Sample B3R received the highest total score (101 points). Sample B3S received 8 points less. The average score of acidified beers was 78.5 points and was higher than that of the nonacidified beers by up to 39.5 points.

Comparison of long-boiled samples with short-boiled samples showed that omitting the long-boiled process results in improved sensory qualities. Gasinski et al. evaluated the quality of beers with mango fruit; beers with added fruit were characterised by better aroma, color, flavour, and overall impression than the control sample [28]. Similarly, Adamenko et al. in their study showed an improvement in organoleptic characteristics when fruit was added to beers [29].

#### Principal Component Analysis (PCA) and Correlation Analysis of the Research Results

**Figure 3:**
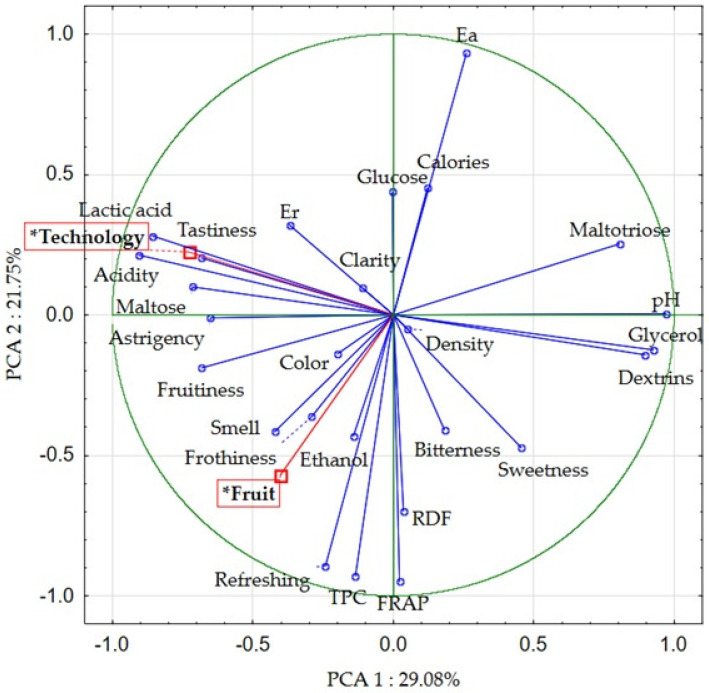
Principal component analysis (PCA) of physicochemical parameters and sensory descriptors of beers. * The names of additional variables used in the PCA analysis are shown in red boxes.

**Figure 4:**
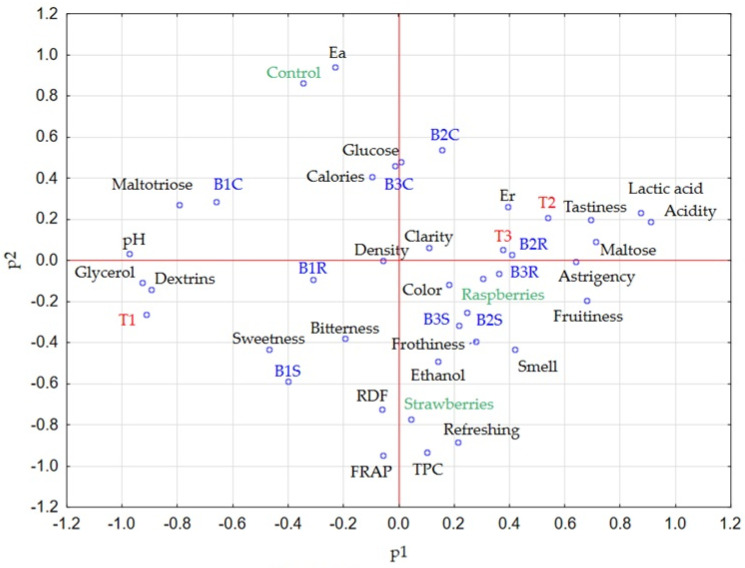
Scatter plot of parameters, analysed samples, fruits ant technologies used in the experiment. (the red text indicates production technologies, green the fruits used, and blue—individual beers).

Principal component analysis (Figure 3 and Figure 4) and correlation analysis (Supplementary Table S3) were performed to enhance the visualization of research findings. The first two principal components (PC) explained 50.83% of the total variation (PC1: 29.08% and PC2: 21.75%). The analysis considered the physicochemical and sensory characteristics of the beers studied, as well as (as additional variables) the type of production technology (T1, T2, T3) and the fruits (raspberries, strawberries) used.

Two additional variables (technology and fruit addition) were used in the PCA analysis. The selection of production technology (the use of acidification and hopping time of the wort) had a significant effect on 14 of the 26 parameters and sensory attributes studied. The key characteristics whose value depended on production technology were the content of lactic acid (r = 0.89) and acidity (r = 0.65), as the well as pH value (r = −0.78) and the glycerol content (r = −0.68). Furthermore, beers made according to T1 technology showed the highest dextrin and glycerol content compared to T2 and T3. The relationship between the content of glycerol and dextrins and the use of the lactic fermentation process was discussed earlier. Figure 4 also illustrates the correlation between T2 and T3 (technologies including acidification) and traits such as palatability, astringency, acidity, lactic acid, and maltose content. There was also a significant correlation between the use of fruit (control, strawberry, raspberry) and 11 of the 26 sensory parameters and traits studied. The most significant of these were refreshing (r = 0.71), colour (r = 0.67) and TPC (r = 0.63), as well as caloric (r = −0.84) and apparent extract (r = −0.65). In particular, the addition of strawberries affected TPC, FRAP and the refreshing sensation, while raspberries affected the colour, frothiness, fruitiness and aroma of beers.

In addition, the observed correlations between the parameters studied. Lactic acid level was positively correlated with acidity (r = 0.82), real extract (r = 0.73), and tastiness (r = 0.61) of the beers. On the other hand, a negative correlation was observed between its content and the pH (r = −0.89) glycerol content (r = −0.81), dextrins (r = −0.68) and the perception of sweet taste (r = −0.58). As the pH of the beers increased, the content of glycerol (r = 0.93), dextrins (r = 0.88) and maltotriose (r = 0.83) increased, while the acidity (r = −0.89), lactic acid content (r = −0.89), maltose (r = −0.67), astringency (r = −0.58), fruitiness (r = −0.56) and tastiness (r = −0.56) decreased. The content of phenolic compounds was positively correlated mainly with FRAP antioxidant activity (r = 0.89) and sensory attributes such as refreshing (r = 0.87) and fruitiness (r = 0.63). The observed correlations between antioxidant activity, polyphenol content, and perceived fruitiness and refreshment likely result from the simultaneous introduction of polyphenols and organic acids via fruit addition [1,2,30]. Fruits improve freshness through their flavor and aroma, while also supplying antioxidant compounds [1,2]. The relation between dextrin content and pH, can be explained by the metabolic activity of *L. plantarum* and the inhibitory effect on aldehyde dehydrogenase at low pH, which we mentioned earlier [18].

## 3. Materials and Methods

### 3.1. Materials and Sample Preparation

#### 3.1.1. Raw Materials

The following malts were used for the production of beer wort: Pilsner malt (Viking Malt, Strzegom, Poland) and wheat malt (Viking Malt, Strzegom, Poland). The beers were hopped with hop varieties: Iunga (Twój Browar, Poland), Marynka (Twój Browar, Poland).

#### 3.1.2. Biological Material

The acidification of the wort was obtained by the lactic acid fermentation process with the *Lactiplantibacillus plantarum strain* WLP693 (Lallemand, Montreal, QC, Canada).

The top fermentation brewer yeast *Saccharomyces cerevisiae* Safale S-33 (Fermentis, Lesaffre, France) was used in the fermentation process of beer wort. Before adding to the wort, the yeast was rehydrated in 10 mL of sterile distilled water at 25 °C for 30 min.

#### 3.1.3. Brewing Technology

Wort was obtained by standard infusion mashing in a stainless steel pot. The mash was made with 50% Pilsner malt (Viking Malt, Strzegom, Polska) and 50% wheat malt (Viking Malt, Strzegom, Polska). The mashing process steps were as follows: 52 °C for 10 min, 62 °C for 30 min, 72 °C for 30 min and 78 °C for 10 min. The wort (24 L) was obtained by filtering the mash and was further processed according to one of the 3 technologies (T1, T2, T3). The wort extract was 8 °Bx (measured using a portable oscillating U-tube density meterDensito 30PX, Mettler Toledo, Columbus, OH, USA) (Analitica EBC 2010). The wort was brought to a boil and then separated into three 6 L samples. Two of the samples were cooled down to 35 °C, the pH was set at 4.5 and inoculated with *L. plantarum*). Lactic acid fermentation was carried out at 30 °C for approximately 48 h, until the pH reached around 3.5. The acidified sweet wort were hopped for 60 min using 7 g of Sybilla variety (60 min) and 5 g of Iunga variety (10 min). Hopping of the non-acidified worts was performed by addition of cold hop extract. The extract was made from acidified wort using boiling time and doses of hops analogous to the remaining samples. Each wort was cooled to 20 °C, the extract was set at 8 °Bx by dilution and inoculated with previously rehydrated *Saccharomyces cerevisiae* Safale S-33 top-fermenting yeast (Fermentis, Lesaffre, France) at a dose of 0.46 g/L according to the manufacturer’s recommendations. Fermentation was carried out at 20 °C for 14 days. Three beers were obtained after primary fermentation. Next, each of the beers was separatde to three samples with a volume of 2 L and frozen fruits were added. We obtained the following: control sample, samples which strawberries and samples which raspberries addition from each of the beers after primary fermentation. The fruits were added at a dose of 10% *w*/*v*. After post-fermentation, the following beer variants were obtained: 3 control beers, 3 strawberry beers and 3 raspberry beers. Post-fermentation was carried out for 14 days at a temperature 20° C. The beers were then filtered to separate the fruit and bottled. For the refermentation process, glucose was used at a dose of 3.5 g/L. The beers were bottled and maturated for 21 days. After maturation, we obtained nine beers: control beers (B1C, B2C, B3C), with strawberry addition (B1S, B2S, B3S) and with raspberry addition (B1R, B2R, B3R) (Table 5).

### 3.2. Analytical Methods

#### 3.2.1. High-Performance Liquid Chromatography (HPLC) Analysis of Carbohydrate, Glycerol, Ethanol and Lactic Acid Content

Carbohydrate profiles (maltose, maltotriose, glucose, dextrin) and basic fermentation products (glycerol, ethanol, and lactic acid) were analyzed by high performance liquid chromatography (HPLC). The samples were degassed and then centrifuged with a laboratory MPW-351R centrifuge (10 min, 5000 rpm). The samples were then diluted two times with water and filtered through 0.22 µm filters. Separation was carried out using a Rezex ROA–Organic Acid H+ (300 × 7.8 mm) column (Phenomenex, Torrance, CA, USA). The HPLC method, using Prominence apparatus (Shimadzu, Kyoto, Japan), was used. Measurement parameters: injection volume—0.02 mL, flow rate—0.6 mL/min, separation temperature—60 °C, mobile phase—0.005 M H_2_SO_4_. Compounds were detected using refractive index detector (RID-10A, Shimadzu, Kyoto, Japan) maintained at 50 °C. Results were presented as g/L of beer.

#### 3.2.2. Total Phenolic Content

The total phenolic content (TPC) in worts and beers was determined using the spectrophotometric Folin-Ciocalteu method [31]. Samples with a volume of 0.1 mL along with 0.2 mL of F-C reagent were placed in the cups using an automatic pipette. After 3 min, 1 mL of 20% m/v Na_2_CO_3_ solution and 2 mL of distilled water were added to each sample. After 1h prepared samples were analyzed using a UV–2401 PC spectrophotometer (Shimadzu, Kyoto, Japan) at the wavelength of 765 nm, distilled water was used as a blind sample. The results were presented as an average value of three repetitions. A calibration curve in the range of 0.30–9.00 mg of gallic acid/L was used to read the results. The results were expressed as mg of GAE per liter of wort or beer.

#### 3.2.3. Ability of Iron Ion Reduction (FRAP)

Analysis of the ability to reduce ferric ions was carried out using the method of Benzie and Strain [32]. The principle of the FRAP method (ferric ion reducing antioxidant parameter) is the reduction of iron-2,4,6-tri(2-pyridyl)-1,3,5-thiazide[Fe(III)-TPTZ)] to the ferric complex at low pH. The FRAP reagent (200 mL) was made by combining 20 mL of the water solution containing 0.1018 g of iron chloride (III) (FeCl_3_) with the 0.0664 g solution of TPTZ (2,4,6-Tris(2-pyridyl)-s-triazine) in 20 mL 40 mmol solution of muriatic acid (HCl) using acetate buffer with pH 3.6. Quantitative analysis was performed using an external standard method using iron (II) sulphate (VI) (2 × 10^−1^ mmol/L) as a reference standard. On this basis, a correlation curve was made between the absorbency value and the compound concentration. The beer (1 mL) was mixed with distilled water and the FRAP reagent (3 mL) was mixed in disposable polystyrene cuvettes. The absorbance was determined using a UV–2401 PC spectrophotometer with wavelength 593 nm and distilled water was used as a blind sample. The results were presented in millimoles of Trolox per litre of wort or beer. The determinations were made in triplicate.

#### 3.2.4. Basic Physicochemical Parameters

The beers were degassed using a 358 A laboratory shaker (Elpin Plus, Lubawa, Poland) and then filtered using paper filters and diatomaceous earth. Next, using an Anton Paar beer analyser DMA 4500 M beer densimeter (Anton Paar GmbH, Graz, Austria), the ethanol content, the real attenuation, the the apparent attenuation, wort extract content and the density of the beer density were measured. The pH value was analysed with a Mettler Toledo F20 m (Mettler Toledo, Columbus, OH, USA).

### 3.3. Sensory Evaluation and Consumer Preferences

The evaluation involved 13 participants, including 7 women and 6 men. Sensory analysis was carried out at the Wrocław University of Environmental and Life Sciences, Faculty of Biotechnology and Food Sciences, in dedicated sensory workstations, which ensured independent evaluations under controlled conditions. The beer samples were coded with numbers from 1 to 9. The scale to assess beer quality descriptors is added as Supplementary Table S1. In addition to descriptive analysis, the panel also performed a hedonic analysis to asses overall preference. All study participants were qualified and experienced in beer sensory analysis. The panel consisted of researchers and students specialising in food technology regularly trained in professional sensory analysis. Participation in the evaluation of products was voluntary. Each participant was informed and agreed to the analysis principles.

Hedonic evaluation of beers was conducted using a ranking method. Thirteen participants were asked to rank beer samples from 1 to 9, where 9 represented the most preferred sample and 1 the least preferred. This method was used to assess overall taste preferences among all samples.

### 3.4. Statistical Analysis

The data obtained were analysed using Statistica 13.5 (StatSoft, Tulsa, OK, USA). One-way variation analysis (ANOVA) with statistical significance level 0.05 was performed. The significance of the differences between the mean values was examined using the Duncan test (*p* < 0.05). Principal component analysis (PCA) was conducted to compare the physicochemical parameters with the sensory characteristics of the beer.

## 4. Conclusions

Research showed that acidification of fruit, and shortening of the boiling time of the wort had a positive effect on both the technological and sensory characteristics of the beers. Beers with fruit addition were characterised by lower caloricity, lower pH value, and lower extract content than other variants. Acidification of the wort resulted in improved sensory characteristics, as well as increased attenuation of dextrins and maltotriose. The use of a shortened boiling resulted in beers with the best sensory characteristics, which makes this technology can be successfully applied in the production of sour beers.

Bioprocessing of food by ethanol and lactic acid fermentation can be an effective method to increase the content of phenolic compounds with antioxidant activity. The levels of these compounds in beer depend not only on the microorganisms used in the fermentation process, but also on the applied technological procedures. Both worts subjected to lactic acid fermentation showed a high phenolic content. The wort produced with a shortened boiling time had more than twice the total phenolic compounds compared to the wort without acidification, hopped with the use of classical technology. The addition of fruits can be an effective method to enrich beers with phenolic compounds and increase their antioxidant activity. The finished strawberry and raspberry beers had had higher total phenolic content compared to samples without fruits, regardless of the acidification and boiling process of the wort. Strawberry beers showed the highest phenolic content and antioxidant potential. The acidification of the wort and the reduced boiling time, despite a beneficial effect on the content of phenolic compounds, did not increase the antioxidant activity of the finished beers. The highest antioxidant capacity was observed for beer made from nonacidified wort produced according to classic technology with the addition of strawberries.

Future studies should investigate the use of other fruits, alternative strains of bacteria and yeast, and different techniques for boiling and hopping, as well as the effect of longer ageing on the key characteristics and functional properties of sour beers. It would also be valuable to investigate the optimisation of fruit doses and the stage of their addition during the brewing process, as these factors can have a significant impact on the extraction of bioactive compounds, fermentation dynamics, and the final sensory profile of beer.

## Figures and Tables

**Table 1:** Basic physicochemical parameters of beers.

Technology	Symbol	Alcohol [%*v*/*v*]	Apparent Extract [%*w*/*w*]	RDF [%]	Density [g/cm^3^]	Calories [kcal/100 mL]	pH
T1	B1C	2.75 ± 0.01 ^1^ f	3.25 ± 0.05 c	57.83 ± 0.05 g	0.9961 ± 0.00 ab	26.16 ± 0.00 d	4.79 ± 0.01 a
	B1S	3.00 ± 0.01 c	2.59 ± 0.01 h	65.30 ± 0.06 b	0.9958 ± 0.00 b	25.36 ± 0.00 f	4.24 ± 0.01 b
	B1R	2.66 ± 0.01 g	2.90 ± 0.01 d	59.99 ± 0.02 d	0.9966 ± 0.00 a	22.92 ± 0.00 g	4.23 ± 0.01 b
T2	B2C	2.76 ± 0.00 f	3.32 ± 0.01 b	58.06 ± 0.55 f	0.9957 ± 0.00 b	27.81 ± 0.00 b	3.70 ± 0.01 d
	B2S	3.08 ± 0.01 b	2.59 ± 0.04 h	65.60 ± 0.23 a	0.9958 ± 0.00 b	25.58 ± 0.00 e	3.66 ± 0.01 e
	B2R	2.52 ± 0.00 h	2.80 ± 0.02 f	63.22 ± 0.21 c	0.9967 ± 0.00 a	21.70 ± 0.00 h	3.56 ± 0.01 g
T3	B3C	2.89 ± 0.01 e	3.38 ± 0.01 a	57.74 ± 0.73 h	0.9957 ± 0.00 b	28.26 ± 0.00 a	3.81 ± 0.01 c
	B3S	3.21 ± 0.01 a	2.74 ± 0.04 g	59.53 ± 0.00 e	0.9956 ± 0.00 b	26.53 ± 0.00 c	3.60 ± 0.02 f
	B3R	2.97 ± 0.00 d	2.86 ± 0.01 e	55.31 ± 0.59 i	0.9959 ± 0.00 b	25.34 ± 0.00 f	3.53 ± 0.01 h

^1^ Values are expressed as mean ± standard deviation (*n* = 2). The values with different letters (a, b, c, d, e, f, g, h, i) within the same column are statistically different (*p* < 0.05). Abbreviations: beers obtained from: T1–non lactic fermented wort, boiled for 60 min, T2–lactic fermented wort, boiled for 60 min, T3–lactic fermented wort, boiled for 5 min; B1C, B2C, B3C–control beers after maturation; B1S, B2S, B3S–strawberry beers after maturation, B1R, B2R, B3R–raspberry beers after maturation, RDF–real degree of fermentation.

**Table 2:** Content of fermentation products and carbohydrate profile of beers.

Technology	Symbol	Glucose [g/L]	Maltose [g/L]	Maltotriose [g/L]	Dextrins [g/L]	Glycerol [g/L]	Lactic Acid [g/L]
T1	B1C	0.28 ± 0.00 ^1^ f	0.00 ± 0.00 d	2.60 ± 0.01 a	23.13 ± 0.01 a	1.62 ± 0.00 a	0.00 ± 0.00 g
	B1S	0.00 ± 0.00 g	0.00 ± 0.00 d	1.08 ± 0.01 c	19.07 ± 0.01 b	1.36 ± 0.00 b	0.00 ± 0.00 g
	B1R	0.76 ± 0.00 a	0.00 ± 0.00 d	0.84 ± 0.00 e	17.64 ± 0.04 c	1.38 ± 0.01 b	0.00 ± 0.00 g
T2	B2C	0.38 ± 0.00 e	0.32 ± 0.01 b	0.58 ± 0.02 g	13.26 ± 0.04 h	1.07 ± 0.04 e	2.08 ± 0.00 d
	B2S	0.00 ± 0.00 g	0.00 ± 0.00 d	0.58 ± 0.00 g	16.17 ± 0.04 f	1.11 ± 0.01 cd	2.21 ± 0.02 b
	B2R	0.53 ± 0.00 b	0.32 ± 0.00 b	0.46 ± 0.01 h	10.73 ± 0.08 i	0.83 ± 0.02 f	1.70 ± 0.01 f
T3	B3C	0.39 ± 0.00 d	0.00 ± 0.00 d	1.39 ± 0.01 b	16.31 ± 0.06 e	1.07 ± 0.03 e	2.14 ± 0.00 c
	B3S	0.00 ± 0.00 g	0.36 ± 0.00 a	0.87 ± 0.00 d	16.71 ± 0.06 d	1.12 ± 0.01 c	2.30 ± 0.01 a
	B3R	0.44 ± 0.00 c	0.31 ± 0.00 c	0.68 ± 0.00 f	13.49 ± 0.01 g	1.08 ± 0.00 de	2.05 ± 0.00 e

^1^ Values are expressed as mean (*n* = 3) ± standard deviation. The values with different letters (a, b, c, d, e, f, g, h, i) within the same column are statistically different (*p* < 0.05). Abbreviations: B1C, B2C, B3C–control beers after maturation; B1S, B2S, B3S–strawberry beers after maturation, B1R, B2R, B3R–raspberry beers after maturation.

**Table 3:** Total polyphenol content (mg GAE/L) of worts and beers.

Technology	Symbol	Worts		Beers		
		Sweet	Hopped	Primary Fermented	Post Fermentated *	Maturated
T1	B1C	39.54 ± 1.64 ^1^ m	84.87 ± 0.92 m	111.55 ± 0.81 l	174.50 ± 1.06 gh	153.56 ± 20.73 ij
	B1S				191.74 ± 2.41 de	237.03 ± 1.96 a
	B1R				126.44 ± 2.25 k	207.24 ± 0.94 bc
T2	B2C	58.27 ± 1.72 n	112.91 ± 0.50 l	176.42 ± 0.30 fgh	150.14 ± 1.06 j	155.24 ± 1.48 ij
	B2S				234.21 ± 1.91 a	212.25 ± 0.86 b
	B2R				163.90 ± 1.96 hij	204.73 ± 0.24 bcd
T3	B3C		188.55 ± 0.60 ef	151.39 ± 0.26 ij	193.80 ± 1.82 cde	165.24 ± 0.73 hi
	B3S				255.02 ± 1.21 a	205.21 ± 0.20 bcd
	B3R				203.63 ± 1.43 bcd	187.79 ± 1.94 efg

^1^ Values are expressed as mean (*n* = 3) ± standard deviation. The values with different letters (a, b, c, d, e, f, g, h, i, j, k, l, m, n) are statistically different (*p* < 0.05). Abbreviations: beers obtained from: T1–non lactic fermented wort, boiled for 60 min, T2–lactic fermented wort, boiled for 60 min, T3–lactic fermented wort, boiled for 5 min; B1C, B2C, B3C–control beers; B1S, B2S, B3S–strawberry beers, B1R, B2R, B3R–raspberry beers; * fruits were added to the beers before post-fermentation.

**Table 4:** Antioxidant capacity FRAP (mmolTE/L of worts and beers).

Technology	Symbol	Worts		Beers		
		Sweet	Hopped	Primary Fermented	Post Fermentated *	Maturated
T1	B1C	0.41 ± 0.01 ^1^ jk	0.63 ± 0.01 gh	0.70 ± 0.01 fg	0.56 ± 0.00 h	0.70 ± 0.02 fg
	B1S				1.17 ± 0.03 a	1.17 ± 0.02 a
	B1R				0.90 ± 0.01 cd	0.85 ± 0.04 de
T2	B2C	0.31 ± 0.00 k	0.46 ± 0.01 ij	0.60 ± 0.00 h	0.61 ± 0.01 gh	0.54 ± 0.02 hi
	B2S				0.98 ± 0.00 bc	0.82 ± 0.01 de
	B2R				0.78 ± 0.01 ef	0.89 ± 0.00 cd
T3	B3C		0.36 ± 0.00 k	0.63 ± 0.01 gh	0.63 ± 0.01 gh	0.56 ± 0.01 h
	B3S				1.06 ± 0.06 bc	1.02 ± 0.02 b
	B3R				0.71 ± 0.02 fg	0.83 ± 0.01 de

^1^ Values are expressed as mean (*n* = 3) ± standard deviation. The mean values with different letters (a, b, c, d, e, f, g, h, i, j, k) are statistically different (*p* < 0.05). Abbreviations: beers obtained from: T1–non lactic fermented wort, boiled for 60 min, T2–lactic fermented wort, boiled for 60 min, T3–lactic fermented wort, boiled for 5 min; B1C, B2C, B3C–control beers; B1S, B2S, B3S–strawberry beers, B1R, B2R, B3R–raspberry beers; * fruits were added to the beers before post-fermentation.

**Table 5:** Technology scheme and sample abbreviations.

Technology	T1			T2			T3		
**Sweet wort**	Non- acidified			Acidified					
**Hopped wort**	Boiled for 60’			Boiled for 60’			Boiled for 5’		
**Primary fermented beers**	Non-acidified young beer			Acidified young beer			Acidified young beer		
**Post-fermented beers**	Control beer	Strawberry beer	Raspberry beer	Control beer	Strawberry beer	Raspberry beer	Control beer	Strawberry beer	Raspberry beer
**Maturated beers**	Control beer (B1C)	Strawberry beer (B1S)	Raspberry beer (B1R)	Control beer (B2C)	Strawberry beer (B2S)	Raspberry beer (B2R)	Control beer (B3C)	Strawberry beer (B3S)	Raspberry beer (B3R)

## Data Availability

Data are available from the corresponding author upon reasonable request.

## Supplementary Materials

The following supporting information can be downloaded at: https://www.mdpi.com/article/10.3390/molecules30163358/s1, Table S1: Scale for assessing beer sensory quality descriptors; Table S2: Result of sensory analysis of beers; Table S3: Correlation analysis of beers physicochemical and sensory parameters.

## References

[1] Gasiński A., Kawa-Rygielska J., Szumny A., Gąsior J., Głowacki A. (2020). Assessment of volatiles and polyphenol content, physicochemical parameters and antioxidant activity in beers with dotted hawthorn (*Crataegus punctata*). Foods.

[2] Kawa-Rygielska J., Adamenko K., Kucharska A.Z., Prorok P., Piórecki N. (2019). Physicochemical and antioxidative properties of Cornelian cherry beer. Food Chem..

[3] Ducruet J., Rébénaque P., Diserens S., Kosińska-Cagnazzo A., Héritier I., Andlauer W. (2017). Amber ale beer enriched with goji berries—The effect on bioactive compound content and sensorial properties. Food Chem..

[4] Cabras I., Bamforth C. (2016). From reviving tradition to fostering innovation and changing marketing: The evolution of micro-brewing in the UK and US, 1980–2012. Bus. Hist..

[5] Bossaert S., Crauwels S., De Rouck G., Lievens B. (2019). The power of sour—A rewiev: Old traditions, new opportunities. Brew. Sci..

[6] Dysvik A., Liland K.H., Myhrer K.S., Westereng B., Rukke E.-O., de Rouck G., Wicklund T. (2019). Pre-fermentation with lactic acid bacteria in sour beer production. J. Inst. Brew..

[7] Mastanjević K., Krstanović V., Lukinac J., Jukić M., Vulin Z., Mastanjević K. (2018). Beer—The Importance of Colloidal Stability (Non-Biological Haze). Fermentation.

[8] Vaughan A., O’Sullivan T., van Sinderen D. (2005). Enhancing the Microbiological Stability of Malt and Beer—A Review. J. Inst. Brew..

[9] Gąsior J., Kawa-Rygielska J., Kucharska A.Z. (2020). Carbohydrates profile, polyphenols content and antioxidative properties of beer worts produced with different dark malts varieties or roasted barley grains. Molecules.

[10] Wiliamson G. (2017). The Role of Polyphenols in Modern Nutrition. Nutr. Bull..

[11] Pojer E., Mattivi F., Johnson D., Stockley S.C. (2013). The Case of Anthocyanin Consumption to Promote Human Health: A Review. Comprevensive Rev. Food Sci. Food Saf..

[12] Dysvik A., La Rosa S.L., De Rouck G., Rukke E.-O., Westereng B., Wicklund T., Ercolini D. (2020). Microbial Dynamics in Traditional and Modern Sour Beer Production. Appl. Environ. Microbiol..

[13] Ciosek A., Rusiecka I., Poreda A. (2020). Sour beer production: Impact of pitching sequence of yeast and lactic acid bacteria. J. Inst. Brew..

[14] Dack R.E., Black G.W., Koutsidis G. (2017). The effect of Maillard reaction products and yeast strain on the synthesis of key higher alcohols and esters in beer fermentations. Food Chem..

[15] Prado R., Gastl M., Becker T. (2021). Aroma and color development during the production of specialty malts: A review. Compr. Rev. Food Sci. Food Saf..

[16] Hwang H., Kim Y.J., Shin Y. (2019). Influence of ripening stage and cultivar on physicochemical properties, sugar and organic acid profiles, and antioxidant compositions of strawberries. Food Sci. Biotechnol..

[17] De Souza V.R., Pereira P.A.P., da Silva T.L.T., De Oliveira Lima L.C., Pio R., Queiroz F. (2014). Determination of the bioactive compounds, antioxidant activity and chemical composition of Brazilian blackberry, red raspberry, strawberry, blueberry and sweet cherry fruits. Food Chem..

[18] Chan M.Z.A., Chua J.Y., Toh M., Liu S.-Q. (2019). Survival of probiotic strain Lactobacillus paracasei L26 during co-fermentation with S. cerevisiae for the development of a novel beer beverage. Food Microbiol..

[19] Wang Z., Zhuge J., Fang H., Prior B.A. (2001). Glycerol production by microbial fermentation: A review. Biotechnol. Adv..

[20] Zhao X., Procopio S., Becker T. (2015). Flavor impacts of glycerol in the processing of yeast fermented beverages: A review. J. Food Sci. Technol..

[21] Onilude A.A., Ayinla G.S., Eluehike C. (2017). Properties of Alpha-amylase of Lactobacillus plantarum Isolated from Cassava Waste Samples. Biotechnol. J. Int..

[22] Yang D., Gao X. (2021). Research progress on the antioxidant biological activity of beer and strategy for applications. Trends Food Sci. Technol..

[23] Nardini M., Garaguso I. (2020). Characterization of bioactive compounds and antioxidant activity of fruit beers. Food Chem..

[24] Everette J.D., Bryant Q.M., Green A.M., Abbey Y.A., Wangila G.W., Walker R.B. (2010). Thorough study of reactivity of various compound classes toward the Folin− Ciocalteu reagent. J. Agric. Food Chem..

[25] Muñoz R., de Las Rivas B., de Felipe F.L., Reverón I., Santamaría L., Esteban-Torres M., Curiel J.A., Rodríguez H., Landete J.M. (2017). Biotransformation of phenolics by *Lactobacillus plantarum* in fermented foods. Fermented Foods in Health and Disease Prevention.

[26] Septembre-Malaterre A., Remize F., Poucheret P. (2018). Fruits and vegetables, as a source of nutritional compounds and phytochemicals: Changes in bioactive compounds during lactic fermentation. Food Res. Int..

[27] Hur S.J., Lee S.Y., Kim Y.C., Choi I., Kim G.B. (2014). Effect of fermentation on the antioxidant activity in plant-based foods. Food Chem..

[28] Gasiński A., Kawa-Rygielska J., Szumny A., Czubaszek A., Gąsior J., Pietrzak W. (2020). Volatile Compounds Content, Physicochemical Parameters, and Antioxidant Activity of Beers with Addition of Mango Fruit (*Mangifera Indica*). Molecules.

[29] Adamenko K., Kawa-Rygielska J., Kucharska A.Z. (2020). Characteristics of Cornelian cherry sour non-alcoholic beers brewed with the special yeast Saccharomycodes ludwigii. Food Chem..

[30] Ritter S., Dölle K., Bargen M., Piatkowski J. (2016). Fruits in Craft Beer: A Study to Evaluate the Impact of Fruits on the pH in the Brewing Process and the Breweries Waste Water. Adv. Res..

[31] Prior R.L., Wu X., Schaich K. (2005). Standardized Methods for the Determination of Antioxidant Capacity and Phenolics in Foods and Dietary Supplements. J. Agric. Food Chem..

[32] Benzie I.F.F., Strain J.J. (1996). The ferric reducing ability of plasma (FRAP) as a measure of “antioxidant power”: The FRAP assay. Anal. Biochem..

