# Supplementary material for: Sour Fruit Beers—Ethanol and Lactic Acid Fermentation in Beer Production
Source: Molecules. 2025 Aug 12;30(16):3358. doi: 10.3390/molecules30163358 (PMC12388021; doi:10.3390/molecules30163358)
Supplement: Supplementary file 1 [file molecules-30-03358-s001.zip › Supplementary Table S1 Scale for assessing beer sensory quality descriptors.pdf]

Supplementary Table S1 Scale for assessing beer sensory quality descriptors

Descriptors	Scale				
	**5**	**4**	**3**	**2**	**1**
**Acidity**	Very sour	Sour	Madium sour	Mildly sour	Non-sour
**Bitterness**	Very bitter	Bitter	Medium bitter	Mildly bitter	Non-bitter
**Sweetness**	Very sweet	Sweet	Medium sweet	Mildly sweet	Non-sweet
**Astrigency**	Very astringent, very tart	Astringent, tart	Medium astringent and tart	Mildly astrigent, non-tart	Non-astrigent, non-tart
**Tastiness**	Very tasty	Tasty	Medium tasty	Mildly tasty	Non-tasty
**Smell**	Very aromatic	Perceptible, pleasant	Medium perceptible, pleasant	Midly perceptible, pleasant	Non-perceptible, non-pleasant
**Clarity**	Very clarity	Clarity	Medium clarity	Mildly clarity	Non-clarity
**Color**	Very bright	Bright	Medium bright	Dark	Very dark
**Frothiness**	Very frothiness	Frothiness	Medium frothiness	Mildly frothiness	Non- frothiness
**Refreshing**	Very refreshing	Refreshing	Medium refreshing	Mildly refreshing	Non- refreshing
**Fruitiness**	Very fruity	Friuty	Medium fruity	Mildly friuty	Non-fruity
