# Supplementary material for: Sour Fruit Beers—Ethanol and Lactic Acid Fermentation in Beer Production
Source: Molecules. 2025 Aug 12;30(16):3358. doi: 10.3390/molecules30163358 (PMC12388021; doi:10.3390/molecules30163358)
Supplement: Supplementary file 1 [file molecules-30-03358-s001.zip › Supplementary Table S2 Result of sensory analysis of beers.pdf]

Supplementary Table S2 Result of sensory analysis of beers

Sample	Acidity	Astrigency	Sweetness	Smell	Tastiness	Clarity	Color	Refreshing	Fruitiness
**B1C**	1.77 ± 0.60	2.23 ± 0.93	2.69 ± 0.75	2.92 ± 0.76	3.08 ± 0.76	3.23 ± 0.83	3.15 ± 0.55	2 ± 0.41	3.15 ± 0.80
**B1S**	2.31 ± 0.85	1.85 ± 0.69	2.38 ± 0.87	4 ± 0.58	2.46 ± 0.52	2.54 ± 0.52	3.38 ± 0.77	4,77 ± 0.44	2.62 ± 0.65
**B1R**	3.08 ± 0.28	2.15 ± 0.69	2.00 ± 0.41	3.77 ± 0.73	3.31 ± 0.63	3.31 ± 0.63	4.54 ± 0.66	4.38 ± 0.65	3.69 ± 0.48
**B2C**	4.69 ± 0.48	2.62 ± 0.65	1.54 ± 0.66	3.08 ± 0.28	3.77 ± 0.60	2.77 ± 0.44	3.00 ± 0.41	1.85 ± 0.38	3.23 ± 0.44
**B2S**	4.08 ± 0.28	2.85 ± 0.69	2.00 ± 0.00	4.23 ± 0.73	4.31 ± 0.63	3.38 ± 0.51	3.62 ± 0.51	4.77 ± 0.44	4.38 ± 0.65
**B2R**	4.85 ± 0.38	3.62 ± 0.51	1.69 ± 0.48	3.77 ± 0.60	4.46 ± 0.52	2.54 ± 0.52	4.38 ± 0.51	4.46 ± 0.66	4.62 ± 0.51
**B3C**	4.08 ± 0.49	2.38 ± 0.51	1.46 ± 0.52	3.08 ± 0.28	2.92 ± 0.49	3.92 ± 0.76	3.62 ± 0.65	2.00 ± 0.00	2.77 ± 0.60
**B3S**	4.15 ± 0.38	2.54 ± 0.52	1.92 ± 0.49	3.92 ± 0.76	3.38 ± 0.51	3.31 ± 0.48	3.62 ± 0.65	4.15 ± 0.69	4.69 ± 0.48
**B3R**	4.15 ± 0.38	3.38 ± 0.65	1.77 ± 0.44	4.15 ± 0.38	4.46 ± 0.52	3.69 ± 0.75	4.77 ± 0.44	4.77 ± 0.44	5.00 ± 0.00

Abbreviations: B1C, B2C, B3C – control beers after maturation; B1S, B2S, B3S – strawberry beers after maturation, BIR, B2R, B3R – raspberry beers after maturation
