# Supplementary material for: Sour Fruit Beers—Ethanol and Lactic Acid Fermentation in Beer Production
Source: Molecules. 2025 Aug 12;30(16):3358. doi: 10.3390/molecules30163358 (PMC12388021; doi:10.3390/molecules30163358)
Supplement: Supplementary file 1 [file molecules-30-03358-s001.zip › Supplementary Table S3 Correlation analysis of beers physicochemical and sensory parameters.pdf]

Supplementary Table S3 Correlation analysis of beers physicochemical and sensory parameters

	Fruit	Technology	TPC	FRAP	Glycerol	Lactic acid	Maltose	Maltotriose	Glucose	Dextrins	Ethanol	RDF	Ea	Er	Density	Calories	pH	Acidity	Bitterness	Sweetness	Astrigency	Tastiness	Smell	Clarity	Color	Frothiness	Refreshing	Fruitiness
Fruit	1,00	0,00	0,63	0,54	-0,29	-0,06	0,26	-0,56	0,37	-0,43	-0,17	0,19	-0,65	-0,53	0,60	-0,84	-0,33	0,25	0,09	0,15	0,38	0,23	0,24	0,00	0,67	0,12	0,71	0,58
Technology		1,00	-0,20	-0,22	-0,68	0,89	0,56	-0,35	-0,11	-0,53	0,44	0,15	0,11	0,58	-0,44	0,39	-0,78	0,65	-0,18	-0,54	0,54	0,46	0,30	0,24	0,00	0,23	-0,07	0,40
TPC			1,00	0,89	-0,09	-0,19	-0,11	-0,47	-0,31	-0,08	0,29	0,54	-0,93	-0,35	0,16	-0,54	-0,16	-0,03	0,32	0,33	0,03	-0,18	0,37	-0,11	0,18	0,33	0,87	0,17
FRAP				1,00	0,11	-0,31	0,03	-0,20	-0,42	0,12	0,36	0,69	-0,87	-0,44	0,09	-0,47	0,02	-0,25	0,34	0,39	0,08	-0,28	0,28	-0,15	0,11	0,29	0,82	0,17
Glycerol					1,00	-0,81	-0,63	0,77	-0,10	0,94	0,04	0,22	0,12	-0,27	-0,03	0,12	0,93	-0,89	0,17	0,49	-0,59	-0,57	-0,29	0,01	-0,11	-0,16	-0,12	-0,50
Lactic acid						1,00	0,59	-0,53	-0,19	-0,68	0,36	-0,07	0,05	0,73	-0,42	0,35	-0,89	0,82	-0,24	-0,58	0,47	0,61	0,38	0,19	-0,11	0,26	-0,09	0,45
Maltose							1,00	-0,51	0,06	-0,69	0,02	0,08	-0,03	0,10	-0,06	-0,06	-0,67	0,61	-0,10	-0,43	0,61	0,42	0,16	-0,03	0,13	-0,03	0,04	0,52
Maltotriose								1,00	-0,09	0,84	-0,06	-0,10	0,46	-0,08	-0,08	0,31	0,83	-0,75	-0,02	0,27	-0,40	-0,34	-0,38	-0,02	-0,24	-0,16	-0,41	-0,44
Glucose									1,00	-0,31	-0,80	-0,53	0,42	-0,52	0,69	-0,43	0,04	0,16	-0,14	-0,14	0,11	0,09	-0,38	0,03	0,59	-0,46	-0,27	0,04
Dextrins										1,00	0,22	0,25	0,10	-0,10	-0,18	0,26	0,88	-0,88	0,16	0,43	-0,60	-0,56	-0,21	0,03	-0,24	-0,05	-0,11	-0,53
Ethanol											1,00	0,78	-0,39	0,59	-0,78	0,53	-0,24	-0,02	0,14	0,00	0,00	-0,08	0,46	0,20	-0,34	0,45	0,30	0,05
RDF												1,00	-0,54	0,01	-0,47	0,17	-0,05	-0,24	0,33	0,21	0,05	-0,37	0,30	0,09	-0,07	0,27	0,51	0,00
Ea													1,00	0,18	-0,10	0,53	0,24	-0,04	-0,26	-0,34	-0,10	-0,05	-0,50	0,05	-0,17	-0,45	-0,90	-0,40
Er														1,00	-0,72	0,74	-0,41	0,36	-0,20	-0,30	0,04	0,40	0,33	0,26	-0,47	0,31	-0,28	0,12
Density															1,00	-0,85	0,17	-0,01	-0,10	0,18	0,08	0,10	-0,20	-0,07	0,57	-0,20	0,16	0,20
Calories																1,00	0,00	-0,05	-0,08	-0,25	-0,16	-0,14	-0,05	0,18	-0,60	0,03	-0,57	-0,38
pH																	1,00	-0,89	0,13	0,48	-0,58	-0,56	-0,42	-0,11	-0,11	-0,26	-0,21	-0,56
Acidity																		1,00	-0,13	-0,58	0,51	0,57	0,27	0,11	0,22	0,26	0,03	0,44
Bitterness																			1,00	0,24	-0,14	-0,28	0,18	-0,19	-0,01	-0,08	0,28	-0,20
Sweetness																				1,00	-0,23	-0,10	-0,02	0,05	-0,01	0,04	0,32	0,01
Astrigency																					1,00	0,45	0,11	-0,09	0,21	0,09	0,22	0,53
Tastiness																						1,00	0,28	0,21	0,17	0,20	0,06	0,74
Smell																							1,00	-0,10	-0,08	0,19	0,46	0,26
Clarity																								1,00	0,15	0,41	-0,15	0,22
Color																									1,00	0,06	0,25	0,42
Frothiness																										1,00	0,32	0,26
Refreshing																											1,00	0,36
Fruitiness																												1,00
